# Multi-Omics Integration for Liver Cancer Using Regression Analysis

**DOI:** 10.3390/cimb46040222

**Published:** 2024-04-19

**Authors:** Aditya Raj, Ruben C. Petreaca, Golrokh Mirzaei

**Affiliations:** 1 Department of Electrical and Computer Engineering, The Ohio State University, Columbus, OH 43210, USA; raj.74@osu.edu; 2 Department of Molecular Genetics, The Ohio State University, Marion, OH 43302, USA; petreaca.1@osu.edu; 3 Cancer Biology Program, The Ohio State University James Comprehensive Cancer Center, Columbus, OH 43210, USA; 4 Department of Computer Science and Engineering, The Ohio State University, Marion, OH 43302, USA

**Keywords:** multi-omics, regression, liver cancer, machine learning

## Abstract

Genetic biomarkers have played a pivotal role in the classification, prognostication, and guidance of clinical cancer therapies. Large-scale and multi-dimensional analyses of entire cancer genomes, as exemplified by projects like The Cancer Genome Atlas (TCGA), have yielded an extensive repository of data that holds the potential to unveil the underlying biology of these malignancies. Mutations stand out as the principal catalysts of cellular transformation. Nonetheless, other global genomic processes, such as alterations in gene expression and chromosomal re-arrangements, also play crucial roles in conferring cellular immortality. The incorporation of multi-omics data specific to cancer has demonstrated the capacity to enhance our comprehension of the molecular mechanisms underpinning carcinogenesis. This report elucidates how the integration of comprehensive data on methylation, gene expression, and copy number variations can effectively facilitate the unsupervised clustering of cancer samples. We have identified regressors that can effectively classify tumor and normal samples with an optimal integration of RNA sequencing, DNA methylation, and copy number variation while also achieving significant p-values. Further, these regressors were trained using linear and logistic regression with k-means clustering. For comparison, we employed autoencoder- and stacking-based omics integration and computed silhouette scores to evaluate the clusters. The proof of concept is illustrated using liver cancer data. Our analysis serves to underscore the feasibility of unsupervised cancer classification by considering genetic markers beyond mutations, thereby emphasizing the clinical relevance of additional global cellular parameters that contribute to the transformative process in cells. This work is clinically relevant because changes in gene expression and genomic re-arrangements have been shown to be signatures of cellular transformation across cancers, as well as in liver cancers.

## 1. Introduction

Cellular transformation and immortalization is a complex process driven primarily by the accumulation of point mutations that change protein sequences [1], but also by other genomic changes, such as structural genomic re-arrangements that produce global changes in chromosomal architecture [2,3]; numerical genomic re-arrangements that modify ploidy [4]; and changes in promoter methylation, which generally affect gene expression patterns [5]. For example, massive and rapid chromosomal re-arrangement events such as chromothripsis have been shown to promote the evolution of certain cancers [6,7]. Although mutation has been primarily used as a genetic signature for cancers [8], these other parameters mentioned above have also been considered when categorizing or analyzing cancers.

Certain cancers are characterized by recurrent chromosomal re-arrangements. For example, the chronic myeloid leukemia (CML) blood cancer is characterized by a reciprocal translocation between chromosomes 9 and 22: t(9;22)(q34;q11) [9,10]. This event fuses the BCR gene on chromosome 22 with the ABL gene on chromosome 9 (BCR::ABL), causing constitutive activation of the ABL kinase, which promotes cell division. Although three different BCR-ABL fusion recombination events have been identified, they all have the effect of removing an N-terminal Abl1 region and replacing it with the serine/threonine kinase domain of BCR [11,12]. This affects an intramolecular interaction within the Abl1 protein required for self-inhibition [13]. Some solid tumors are also characterized by recurrent re-arrangements, and many have been used for cancer detection, prognostication, and prediction [14,15,16]. Recently, the advent of next-generation sequencing (NGS) technology has detected numerous other chromosomal re-arrangements, but whether these are recurrent or can be used to classify cancers is an active area of research. We have previously shown that it is possible to classify cancers using chromosomal re-arrangement data [3,17]. In this report, we integrate chromosomal rearrangements with other omics to generate more complete cancer classification models.

Gene expression and methylation changes have also emerged as cancer signatures and can be used for the classification of cancers [18,19]. Liver chances have also been shown to be characterized by unique changes in gene expression and chromosomal re-arrangements [20,21]. Similarly, gene expression [22,23] and promoter methylation [5] profiles have been used to delineate unique cancer signatures.

NGS technology has allowed for high-throughput and rapid data generation for genomes, epigenomes, transcriptomes, proteomes, metabolomes, and phenomes. There is an objective in the bioinformatics field to correlate multiple genetic and genomic events, especially using large pan-cancer analysis, with the goal of generating a more comprehensive map of tumor formation and evolution [24]. It stands to reason that integration of several omics allows for simultaneous analysis of the human genome at multiple levels of complexity, as well as the extraction of increasingly more unique and accurate cancer signatures. Additionally, multi-omics data integration across different functional levels provides a better understanding of the underlying biology of cancer [25]. Multi-omics data have already been used in regression, classification, and clustering models with varying predicting outcomes [25,26,27,28,29]. For example, in a study by Capper et al., DNA methylation was used for the classification of nervous system tumors, demonstrating its application in a routine diagnostic practice [30]. Similarly, DNA methylation was utilized in cancer classification for sinonasal tumors [31]. Yu et al. [32] used copy number variant as a biomarker for lung cancer diagnosis. Bluszek et al. classified chordomas using DNA methylation and RNA sequencing [33], and Wang et al. developed a prognostication tool for ovarian cancer [34]. These few recent examples demonstrate that there is a biological basis for using multi-omics in cancer classification. The integration of multi-omics has been explored in previous studies [35], emphasizing the ongoing investigation into this approach.

Current machine learning techniques based on multi-omics data integration have been reviewed in previous literature [36,37]. Specifically, a multi-omics integration using mRNA expression, DNA methylation, and microRNA expression data was proposed using a graph convolutional neural network [38]. Similarly, a gradient-boosting classifier [32] was proposed by Yu et al. to classify lung cancer using copy number variants. We have also developed reinforcement-learning-based omics integration for liver cancer. A complementary review of machine learning techniques using gene expression data is provided in [39].

In this study, we investigate the contributions of CNV (copy number variant), gene expression, and DNA methylation (DNA-met) to the classification of cancer and normal samples in liver hepatocellular cancer (LIHC). Specifically, we examine the extent to which each variable contributes to the classification results. To achieve this, we employed supervised (regression) and unsupervised (clustering) learning techniques. We used regression to derive the optimal formulation based on a statistical significance study using p-values of coefficients. We employed k-means clustering to detect two clusters: tumor tissue and normal tissue. We computed performance metric silhouette scores to measure the quality of the clusters generated and used p-values to determine which formulation was significant for the integration of the omics data. The novelty of the work can be summarized in three major points: (1) This study introduces an interpretable integration strategy, as opposed to a black-box, neural-network-based technique, for omics integration; (2) the approach employed is simple yet efficient, utilizing a combination of regression and clustering to identify the most significant formulation for integrating omics data; and (3) the study demonstrates that LIHC can be categorized by genomic changes beyond mutations.

The approach outlined in the article serves as an alternative method to traditional imaging for defining tumor and normal samples. There are several benefits of using multi-omics for prediction of normal and cancer samples. First, multi-omics data may capture molecular changes at an earlier stage than imaging, enabling early detection of abnormalities before they manifest as visible changes in imaging modalities. Second, tumors often exhibit molecular heterogeneity, where different regions of a tumor may have distinct molecular profiles. Clustering with multi-omics data allows for a better understanding of intra-tumor heterogeneity, guiding treatment strategies that account for diverse molecular characteristics within a single tumor. For example, as tumors progress, they are known to acquire resistance to drugs that target specific enzymes (small molecule inhibitors), and a more comprehensive view of tumor genomic and genetic changes can aid in better drug design to counteract various resistance mechanisms [40,41].

## 2. Materials and Methods

### 2.1. Data Processing

The LIHC dataset was downloaded from the TCGA dataset (https://www.cancer.gov/tcga (accessed on 9 September 2022)) using TCGA Assembler R package. Data include copy number variations (CNV), gene expression (RNA-seq), and DNA methylation (DNA-met) for both primary tumors and normal controls. The DNA-met data were generated using the methylation-450 platform and the Infinium HumanMethylation450 Beadchip assay. The CNV data were collected using the cna_cnv.hg19 platform and Affymetrix SNP array 6.0 assay. RNA-seq data were acquired using the gene.normalized_RNA-seq platform and the Illumina HiSeq assay. The total number of samples is shown in Supplementary Table S1.

For DNA-met data, we calculated the average methylation values by mapping CpG islands within 1500 bps from the transcription start site (TSS) (both DNAse hypersensitive and hyposensitive). We identified the samples (patient identifiers) that had complete information for all three omics (CNV, RNA-seq, and DNA-met) and discarded samples with missing data for any of these omics. Genes with more than 20% missing values across all samples and samples with more than 20% missing values across the genes were removed. We performed max–min normalization on CNV, DNA-met, and log-transformed RNA-seq data to bring each omics into a common scale ranging from 0 to 1. This preprocessing was carried out using the R programming language. After preprocessing, there were 18,038 genes for 39 samples belonging to the normal tissue class and 18,045 genes for 364 samples in the tumor tissue class. To ensure a balanced approach for multi-omics integration within regression models, we selected an equal number of samples (39 samples) from both classes. These 39 samples were randomly chosen from a total of 364 tumor tissue samples. However, the entire dataset was utilized for clustering analysis.

**Figure 1:**
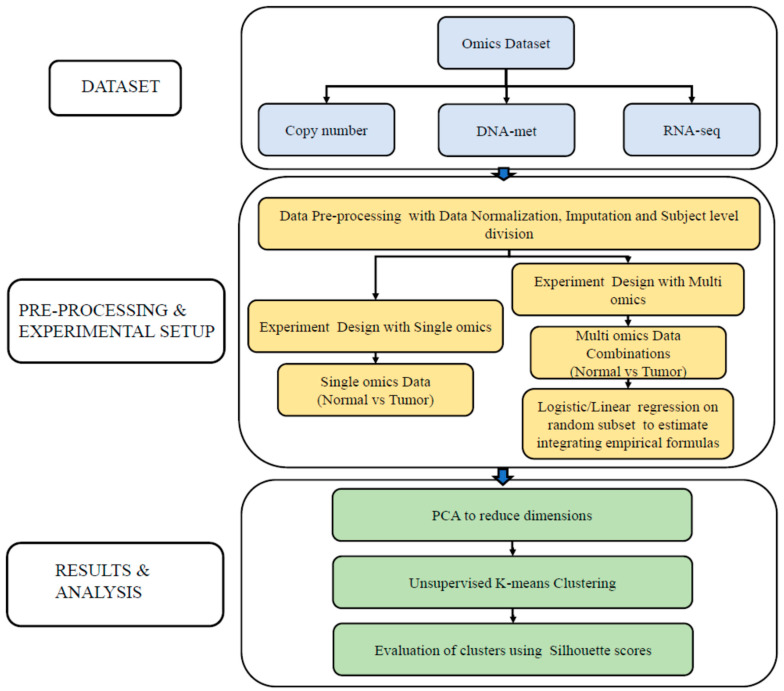
The multi-omics analysis framework. Data were obtained from TCGA and pre-processed. The analysis was conducted in both single-omics and multi-omics divisions. In single-omics division, each of the omics (CNV, DNA-Met, and RNA_seq) was processed individually, with PCA for dimension reduction and k-means clustering. In the multi-omics division, the omics were integrated in various combinations using a regression model, followed by PCA and clustering.

We conducted data analysis involving subject-level data division. The overall process is shown in Figure 1. We performed two sets of analyses: (1) single-omics, aiming to understand the contribution of each omics to normal and tumor classification separately; and (2) multi-omics, to understand the extent to which each omics contributes to optimal classification accuracy by integrating the three omics. For the multi-omics analysis, we especially conducted regression to derive an interpretable formula. In both the sets of single and multi-omics analyses, we performed principal component analysis (PCA) [22] for dimension reduction of the features followed by k-means clustering to classify the samples into normal and tumor groups. The details of single-omics and multi-omics analyses are outlined below.

### 2.2. Single-Omics Analysis

In single-omics analysis, we examined CNV, RNA_seq, and DNA-met data individually and performed k-means clustering with k set to 2 to group normal and tumor samples into two clusters. Additionally, we conducted PCA on a combined dataset to reduce the dimensions of samples within each class to a set number of features equal to the number of PCs. PCA allows us to minimize correlations while retaining most of the total variation present in the data. During dimensionality reduction, we projected the data onto a selected number of principal components, which, in this work, was set to two components (PC = 2, since this achieves the best clusters). These principal components are essentially the eigenvectors of the covariance matrix computed from the data. The choice of the number of principal components is guided by the eigenvalues associated with these eigenvectors, with higher eigenvalues capturing a larger percentage of the total data variance. After this reduction, we combined the data from the two classes and transposed them. This final integrated dataset resulted in matrices $X_{1},\;X_{2},\;X_{3}$ for CNV, RNA-seq, and DNA-met, respectively, each with dimensions m×n. Here, m represents the total number of samples, and n is the total number of features. Each of these matrices was subsequently used as input for the unsupervised clustering algorithm, independently.

### 2.3. Multi-Omics Analysis

In the multi-omics analysis, we applied the same preprocessing steps as described above for single-omics data. The integration of omics data was conducted with the aim of identifying the most effective combinations of RNA-seq, DNA-met, and CNV data. Our objective was to establish an equation in the form of:

(1) y=f(CNV,RNA−seq,DNA−met)

where y is the integrated features; f represents a mapping function (which can be linear or non-linear); and CNV, RNA-seq, and DNA-met serve as predictors. We conducted linear and logistic regression analyses to compute the coefficients in Equation (1) and identified the linear and nonlinear relationships between the dependent variable (overall classification) and the predictors (omics). To assess the statistical significance of individual omics data, we calculated p-values and examined the p-values of the coefficients associated with each predictor. A lower p-value (less than 0.05) indicated that the predictors had a non-zero impact on the dependent variable. Conversely, higher p-values suggested that the predictors could independently influence the dependent variable, and each predictor could be used individually to predict its value. Additionally, these coefficients with their p-values were computed for each gene (18,000+) individually extracted from regression analysis. We maintained a running average of the coefficients and p-values computed to obtain the final estimates.

When using a linear function, the omics data are integrated as a linear combination with coefficients $\alpha_{0}$, $\alpha_{1}$, and $\alpha_{2}$, as shown in Equation (2). These coefficients are estimated using linear regression.

(2) $$y=\alpha_{0}(CNV)+\alpha_{1}(DNA-met)+\alpha_{2}(RNA-seq)$$

For non-linear function, we used logistic regression to compute the coefficients $\beta_{0}$, $\beta_{1},$ and $\beta_{2}$ as:

(3) $$y=1+exp{\left(-\beta_{0}\left(CNV\right)-\beta_{1}\left(DNA-met\right)-\beta_{2}(RNA-seq)\right)}^{-1}$$

The objective function for both linear and logistic regressions aimed to minimize the least square error.

Once these relationships were computed, we combined the omics data and performed clustering, following a similar approach as that used with single-omics data. Clustering was executed based on the linear and logistic regression results, emphasizing the significant p-values obtained for the best combination of multi-omics data. We utilized k-means clustering (k=2) to classify samples into two groups: tumor samples and normal controls.

To integrate multi-omics data, we explored various combinations of these omics, as outlined in Supplementary Table S2. Our experiments involved both linear and logistic regressions applied to all possible combinations of the three omics datasets. Each experiment generated ∑i=1nni set of models, where n represents the total number of omics (i=1,2,3). For instance, when considering CNV and RNA-seq as predictors, we created three distinct models: (1) CNV alone, (2) RNA-seq alone, and (3) the integration of CNV and RNA-seq. All these models from each experiment were employed for clustering, and we assessed their prediction scores and statistical significance. The table presents only those models that generated separate clusters. For instance, in the linear regression based on CNV and RNA-seq, only one model out of the three achieved separated clusters (y=0.17(RNA−seq)).

Notably, not all models were statistically significant. The following combinations, however, produced significant *p*-values:

(1) The combination of CNV and DNA-met data led to the following relationship:

- Linear regression:
  (4) y=0.3(CNV)+3.3(DNA−met)

Significant *p* values ($p_{\alpha_{0}}=0.001$, $p_{\alpha_{1}}=0.001$) were achieved for the coefficients $\alpha_{0}=0.317$ and $\alpha_{1}=3.34$ for CNV and DNA-met, respectively.

• Logistic regression:
  (5) $$y={\left(1+\exp(0.9\left(CNV\right)+61.9\left(DNA-met\right))\right)}^{-1}$$

Significant p-values ($p_{\beta_{0}}=0.004$, $p_{\beta_{1}}=0.004$) were achieved for the coefficients $\beta_{0}=-0.997$ and $\beta_{1}=-61.95$ for CNV and DNA-met, respectively.

(2) The combination of RNA-seq and DNA-met data using the linear regression resulted in the equation:
  (6) y=0.3(RNA−seq)+3.7(DNA−met)

Significant p-values ($p_{\alpha_{2}}=0.0003$, $p_{\alpha_{1}}=0.001$) were achieved for the coefficients $\alpha_{2}=0.379$ and $\alpha_{1}=3.756$ for RNA-seq and DNA-met, respectively. The optimal combinations of the three omics with significant p-values yielded well-separated clusters for clustering tumor and normal samples (see Section 3).

We further implemented an autoencoder (neural-network-based approach) to integrate the 3-omics data. An autoencoder accomplishes the reconstruction of its input features through a nonlinear transformation of the original features. In this process, the autoencoder generates new nonlinear features from its original input feature set. Autoencoders have the ability to automatically learn nonlinear features from unlabeled data by setting the output value as equal to the input value. The developed autoencoder had a depth of 2, and its architecture is detailed in Supplementary Table S3. During training, the model underwent 50 epochs with a batch size of 8 and utilized the Adam optimizer with a learning rate of 0.0001. Initially, each sample possessed 18,038 features, accounting for both normal and cancer data (comprising each of CNV, RNA-seq, and DNA-met). Dimensionality was reduced to 403 features using PCA, capturing 99% of the total variability in the data. The omics data for each sample were concatenated, resulting in a combined feature dimension of 1209 (3 × 403), which served as input for the encoder’s fully connected layers. PCA was performed for dimensionality reduction due to the significant computational complexity associated with a feature dimension of 54,114 (18,038 × 3) when utilizing a fully connected network with an equivalent number of nodes. The output of the encoder produced integrated omics corresponding to each sample, forming the latent space. Subsequently, the latent space vectors were fed into the decoder, and the mean squared error (MSE) was employed as the loss function for training the autoencoder model. Following training, the latent space vectors were extracted, and clustering was performed. Additionally, we conducted clustering based on stacking, which simply integrated the CNV, DNA-met, and RNA-seq features by concatenating them into a joint matrix (see Supplementary Table S4). Stacking simply consists of concatenating each omics into a single feature.

## 3. Results

The primary objective of this study was to categorize samples with subject-level division into two distinct groups: normal samples and tumor samples. To achieve this, we developed regression models followed by unsupervised learning using the k-means algorithm for clustering the samples, which initiates by randomly selecting k centroids from the available data points. Subsequently, it assigns observations to these k clusters in a manner that minimizes the sum of squares between the observations and the centroid features within each cluster. In this specific case, we set the number of clusters to two, corresponding to the tumor and normal classes. To investigate the impact of these omics data on sample clustering, we conducted separate analyses for each of these three elements independently and in combination, as described in the Section 2 (Supplementary Table S1). We evaluated the performance of the models using three metrics (**1**) probability score: ratio of experiments in which a particular combination resulted in well-separated clusters to the total number of possible experiments; (**2**) significant p-values from regression: significance of the relationships between CNV, RNA-seq, and DNA-met in the model and the clustering outcome; and (**3**) silhouette scores.

**Figure 2:**
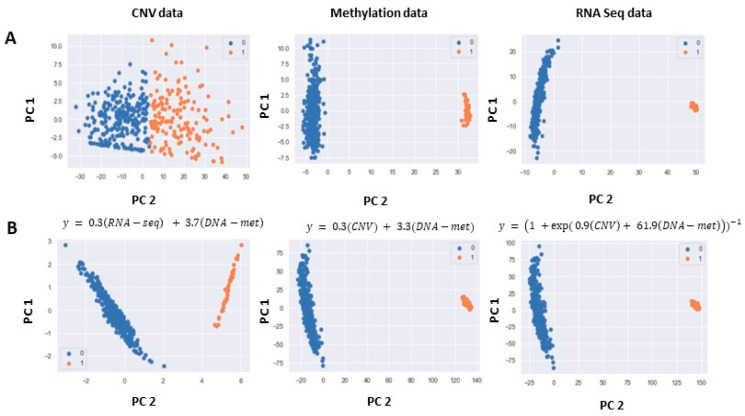
Clustering of cancer versus normal samples using single-omics and multi-omics in LIHC. Clusters labelled as 1 represent tumor samples, and clusters labelled as 0 represent normal samples. Features are extracted using PCA. Results are shown based on two principal components (PC1, and PC2) (**A**) Clustering using single-omics divisions of CNV data, DNA-met data, and RNA-seq data. (**B**) Clustering based on optimized regression models with significant p-values.

In single omics analysis, the RNA-seq data exhibited well separated clusters. In DNA-met, the clusters followed a similar pattern as RNA-seq. However, it is important to note that the intra-cluster variance for normal samples was higher in DNA-met compared to RNA-seq. Lower variance is indicative of a more favorable cluster output. On the other hand, in CNV data, the clusters were not well separated (Figure 2A). In multi-omics analysis, we identified the optimal combination of omics data by deriving optimized regression models to classify tumor and normal samples, as detailed in the Section 2. We then applied PCA and extracted different PCs for dimension reduction, followed by k-means clustering. Figure 2B illustrates the clusters generated using the derived and optimized models. Notably, we obtained significant p-values for two specific combinations: (1) the integration of CNV and DNA-met data and (2) the integration of RNA-seq and DNA-met data. These results show that, even in the absence of multi-omics integration, genomic data other than mutation can differentiate cancer genomes from normal genomes.

In addition, we calculated a probability score, i.e., the ratio of experiments in which a particular combination resulted in well-separated clusters to the total number of possible experiments (details in Section 2). This analysis allowed us to assign probability scores to various scenarios. It is important to note that these scenarios were not limited to statistically significant integrations. In the analysis, a score of 1 indicates that the corresponding combination consistently produced well-separated clusters in all experiments, regardless of its statistical significance. Notably, our observations revealed that utilizing CNV data alone had a 0.16 probability of producing well-separated clusters. This probability significantly increased to 0.5 when integrated with RNA-seq and further improved to 0.75 when combined with DNA-met data. The results of the regression analysis, including significant p-values and probability scores, are presented in Table 1. These outcomes were derived from the optimized regression models as explained in Section 2 and Supplementary Table S1. Notably, these models, followed by clustering, successfully categorized the samples. However, it is crucial to note that not all omics combinations exhibited significant p-values and high probability scores. Specifically, the analysis revealed that RNA-seq and DNA-met stood out with the highest probability scores, accompanied by significant p-values. Subsequently, the integration of CNV and DNA-met yielded a probability score of 0.75. Importantly, this combination also maintained a significant p-value. These findings underscore the performance of various omics integrations in the classification task, emphasizing the importance of considering statistical significance.

**Figure 3:**
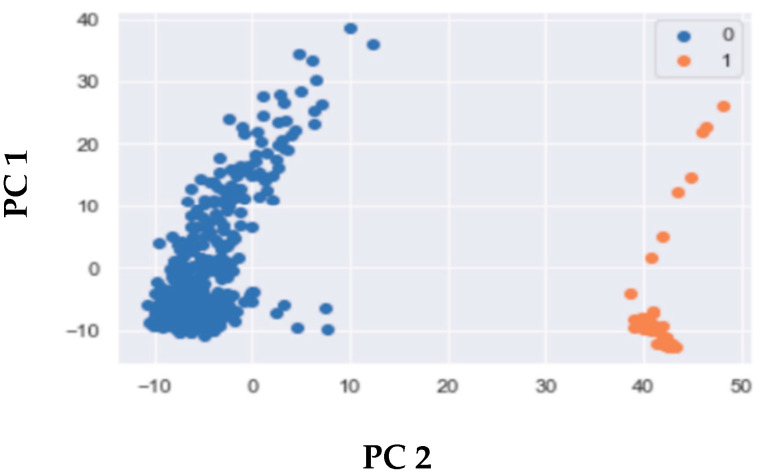
Clustering of normal vs. cancer sample based on autoencoder-based integration. Autoencoders have the capability to automatically learn nonlinear features from unlabeled data by setting the output value as equal to the input value. The autoencoder successfully produced well-separated clusters for multi-omics integration. Clusters labeled as 0 represent tumor samples, and clusters labeled as 1 represent normal samples.

**Figure 4:**
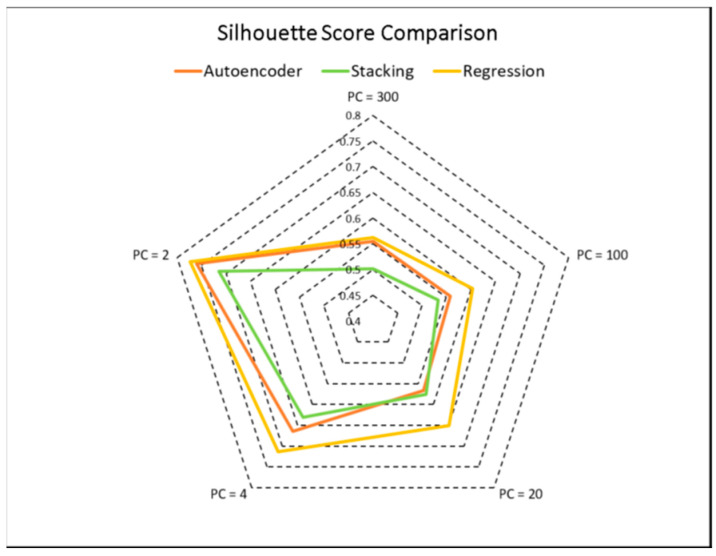
Silhouette score comparison among autoencoders, stacking, and regression-based omics integration. The clusters obtained through the regression and autoencoder methods show well-separated and dense clusters (high silhouette score) with PC = 2.

The results of clustering, based on the autoencoder, are illustrated in Figure 3. Also, model efficacy was evaluated using silhouette scores, and the results were compared across different integration techniques, as displayed in Figure 4. Our results revealed that the integration of CNV and DNA-met data using regression (Equations (4)–(6)) yielded higher silhouette scores with fewer principal components compared to stacking and autoencoders.

## 4. Discussion

In this study, utilizing liver cancer data, we demonstrated the effectiveness of CNV, methylation, and gene expression (RNA-seq) data in terms of accurately characterizing cancer. We identified regression models that enable the reliable clustering of tumor and normal samples through RNA-seq, DNA methylation, and CNV, as well as their integration, achieving significant p-values. Our experiments with single-omics data reveal that, based on our proposed models, CNV alone cannot distinctly separate the two classes into well-separated clusters. However, RNA-seq and DNA-methylation are individually capable of accurately identifying tumor and normal samples with high precision. This is not unexpected, because promoter methylation controls gene expression [42]. Consequently, changes in methylation patterns should correlate strongly with changes in gene expression, as they drive gene expression [43], which is precisely what we found. Various hepatocellular carcinoma studies have shown that methylation of certain gene promoters alters gene expression (e.g., [44,45,46,47,48]). The analysis presented in this paper shows that this strong correlation between this epigenetic mark and gene expression can be used as a characterization tool for liver cancers.

Copy number variations are driven by global chromosomal re-arrangements. Although some level of chromosomal instability is present in all cancers, it only drives the evolution of certain cancers, such as blood and prostate cancers [6,49]. These re-arrangements juxtapose unrelated genomic loci, causing changes in gene expression [50,51,52]. Although we and others have shown that certain re-arrangements do occur in liver cancers [3,53], the present study shows that they may not be sufficiently abundant to be used as a classification tool. However, the integration of RNA-seq with DNA-methylation, as well as CNV with DNA-methylation, yields the most significant p-values with good probability scores. Notably, the combination of RNA-seq, DNA-methylation, and CNV, while providing high probability scores, does not reach statistical significance. Further, the proposed integration strategy performs better than neural-network-based integration in terms of silhouette scores while remaining interpretable and reproducible, with distinct empirical formulas.

The analysis presented in this study highlights the importance of omics other than mutation in classifying cancers. Although liver cancer signatures have previously been characterized by mutation [8], we demonstrate that other global genomic processes can be used in classifying cancers, either by complementing mutation data or, in some cases, on their own. Thus, at least in the case of liver cancer, it is possible to classify and possibly diagnose disease using omics other than mutation. Further, the observation that, even with limited available data, we observed significant differences between control (normal tissues) and cancer samples suggests that these omics are clinically relevant and may have implications for the prognostication or treatment of cancers.

## 5. Conclusions

In this study, we utilize liver cancer data to showcase the effectiveness of CNV, methylation, and RNA-seq data in accurately characterizing cancer. Regression models identify reliable clustering of tumor and normal samples through these omics, achieving significant *p*-values. While CNV alone fails to distinctly separate classes, RNA-seq and DNA methylation individually identify samples with high precision, reflecting the correlation between methylation patterns and gene expression. Additionally, integrating RNA-seq with DNA methylation or CNV with DNA methylation yields significant *p*-values. Our findings underscore the importance of considering omics beyond mutation in cancer classification, suggesting their clinical relevance in prognosis and treatment. As more data become available, these omics will prove to be increasingly actionable in clinical settings.

**Study Limitations**. Omics data integration typically involves matching samples across multiple omics, leading to the limitation of a smaller number of samples with multi-omics data. This constraint can potentially impact machine learning performance, as the reduced sample size may affect the robustness and generalizability of the models. Additionally, when data sets are small, correlations within one set of data may be different from another set. However, as the size of the dataset increases, a more holistic picture will emerge. The work presented in this paper is not meant to be used in a clinical setting. Rather, we show that it is possible to integrate multiple omics even with small datasets. It stands to reason that an increase in the dataset’s size would likely yield clinically actionable conclusions.

It is, therefore, important to consider and address this limitation when interpreting the results of the analysis, and to explore strategies for mitigating its potential impact on the overall study outcomes. Additionally, this study assumes no or little correlation between the three omics data used in this work, which further influences the interpretation of the integrated results and warrants careful consideration in the context of the study’s findings. Finally, other confounding factors, such as tumor purity, are not considered here because such data were not available on COSMIC. As more data become available, these variables could be integrated into these analyses to generate a more holistic cancer genomics map.

## Figures and Tables

**Table 1:** Analysis results for multi-omics integration for classification of normal and cancer samples for LIHC dataset. ${p_{\alpha_{0}},\;p}_{\alpha_{1}}$, $p_{\alpha_{2}}$, $p_{\beta_{0}}$, $p_{\beta_{1}},\;and\;p_{\beta_{2}}$ represent the *p*-values corresponding to CNV (0), DNA-met (1), and RNA seq (2) for linear (α) and logistic regressions (β). The derived optimized combination demonstrates a high prediction score; however, not all of these combinations exhibited statistical significance.

Omics	Significant p-Value	Probability Score [0, 1]
**RNA-seq + DNA-met** (Equation (6))	Yes ${(p}_{\alpha_{2}}$: 0, $p_{\alpha_{1}}$: 0.001)	1
**CNV + DNA-met** (Equations (4) and (5))	Yes ${(p}_{\alpha_{0}}=$ 0.001, $p_{\alpha_{1}}=$ 0.001) ${(p}_{\beta_{0}}=$ 0.004, $p_{\beta_{1}}=$ 0.004)	0.75
CNV + RNA-seq (Optimized models presented in Supplementary Table S1)	No	0.5
CNV + RNA-seq + DNA-met (Optimized models presented in Supplementary Table S1)	No	1

## Data Availability

The codes underlying this article are available in Github and can be accessed with https://github.com/AR13ar/MultiOmics-Integration (accessed 18 April 2022). The dataset used and analyzed during the current study is available from TCGA Research Network: https://portal.gdc.cancer.gov/ (accessed 18 April 2022).

## Supplementary Materials

The following supporting information can be downloaded at: https://www.mdpi.com/article/10.3390/cimb46040222/s1, Table S1: Total number of samples for tumor and control; Table S2: Different combination of omics data using linear and logistic regression models; Table S3: Autoencoder architecture; Table S4: Silhouette score and variance of Autoencoder and random stacking using regression analysis with different number of PCs.
