# Supplementary material for: Multi-Omics Integration for Liver Cancer Using Regression Analysis
Source: Curr Issues Mol Biol. 2024 Apr 19;46(4):3551–62. doi: 10.3390/cimb46040222 (PMC11049490; doi:10.3390/cimb46040222)
Supplement: Supplementary file 1 [file cimb-46-00222-s001.zip › cimb-2899419-supplementary.pdf]

Supplementary Table S1. Total number of samples for tumor and control.

**Type**	**RNA-seq**	**DNA-met**	**CNV**
Tumour	371	377	371
Control	52	52	86

Supplementary Table S2. Different combination of omics data using linear and logistic regression models.

Regression Type	Coefficients	P-values	Derived Empirical Formula
Linear	α_0 : 0.607<br>α_2 : 0.17	p_{α_0}: 0.331<br>p_{α_2}: 0.52	Not statistically significant integration<br>$y = 0.17\,(RNA - seq) + 0.607\,(CNV)$<br>$y = 0.17\,(RNA - seq)$
Logistic	β_0 : 0.887<br>β_2 : -2.607	p_{β_0}: 0.475<br>p_{β_2}: 0.219	Not statistically significant integration<br>$y = (1 + \exp(-0.8(CNV)))^{-1}$<br>$y = (1 + \exp(2.6\,(RNA - seq)))^{-1}$<br>$y = (1 + \exp(-0.8\,(CNV) + 2.6(RNA - seq)))^{-1}$
Linear	α_0 : 0.317<br>α_1 : 3.34	p_{α_0}: **0.00161**<br>p_{α_1}: **0.00129**	Statistically significant integration<br>$y = 0.3(CNV) + 3.3(DNA - met)$<br>$y = 3.3(DNA - met)$
Logistic	β_0 : -0.997<br>β_1 : -61.959	p_{β_0}: **0.00456**<br>p_{β_1}: **0.00482**	Statistically significant integration<br>$y = (1 + \exp(0.9(CNV) + 61.9(DNA - met)))^{-1}$<br>$y = (1 + \exp(61.9(DNA - met)))^{-1}$
Linear	α_2 : 0.379<br>α_1 : 3.756	p_{α_2}: 0.000379<br>p_{α_1}: 0.001057	Statistically significant integration<br>$y = 0.3(RNA - seq) + 3.7(DNA - met)$<br>$y = 0.3(RNA - seq)$<br>$y = 3.7(DNA - met)$
Logistic	β_2 : -40.8<br>β_1 : 154.2	p_{β_2}: 0.152<br>p_{β_1}: 0.152	Not Statistically Significant integration<br>$y = (1 + \exp(40.8(RNA - seq) - 154.2(DNA - met)))^{-1}$<br>$y = (1 + \exp(-154.2(DNA - met)))^{-1}$<br>$y = (1 + \exp(40.8(RNA - seq)))^{-1}$
Linear	α_0 : 0.317<br>α_2 : -0.005<br>α_1 : 4.081	p_{α_0}: 0.1926<br>p_{α_2}: 0.137<br>p_{α_1}: 0.000507	Not Statistically Significant integration<br>$y = 0.317(CNV) - 0.005(RNA - seq) + 4.081(DNA - met)$<br>$y = 0.317(CNV) + 4.081(DNA - met)$<br>$y = -0.005(RNA_{Seq}) + 4.081(DNA - met)$<br>$y = -0.005(RNA - seq)$<br>$y = 4.081(DNA - met)$
Logistic	β_0 : 12<br>β_2 : -47.2<br>β_1 : 81.4	p_{β_0}: 0.29<br>p_{β_2}: 0.154<br>p_{β_1}: 0.152	Not Statistically Significant integration<br>$y = (1 + \exp(-12(CNV) + 47.2(RNA - seq) - 81.4(DNA - met)))^{-1}$<br>$y = 1/(1 + \exp(-12(CNV) + 47.2\,(RNA - seq)))^{-1}$<br>$y = 1/(1 + \exp(47.2(RNA - seq) - 81.4(DNA - met)))$<br>$y = (1 + \exp(47.2(RNA - seq)))^{-1}$<br>$y = (1 + \exp(-81.4(DNA - met)))^{-1}$

Supplementary Table S3. Autoencoder architecture.

Encoder		Decoder	
Fully connected	(1209, 512)	Fully connected	(400, 512)
Fully connected	(512, 400)	Fully connected	(512, 1209)

Supplementary Table S4. Silhouette score and variance of Autoencoder and random stacking using regression analysis with different number of PCs.

Autoencoder		
**Principal Component**	**Variance**	**Silhouette Score**
PC = 2	0.6305	0.7602
PC = 4	0.7655	0.6643
PC = 20	0.9571	0.5664
PC = 100	0.9946	0.5576
PC = 300	0.9999	0.5554
Random Stacking		
PC=2	0.6040	0.7144
PC=4	0.7160	0.6311
PC=20	0.8128	0.5753
PC=100	0.9065	0.5326
PC=300	0.9838	0.5017
